# What is the burden of asymptomatic coronavirus infections?

**DOI:** 10.1016/j.nmni.2023.101101

**Published:** 2023-02-15

**Authors:** Jaffar A. Al-Tawfiq, Ziad A. Memish, Kauthar J. Altawfi, Qiuwei Pan, Patricia Schlagenhauf

**Affiliations:** Infectious Disease Unit, Specialty Internal Medicine, Johns Hopkins Aramco Healthcare, Dhahran, Saudi Arabia; Division of Infectious Diseases, Indiana University School of Medicine, Indianapolis, IN, USA; Division of Infectious Diseases, Johns Hopkins University, Baltimore, MD, USA; King Saud Medical City, Ministry of Health, Riyadh, Saudi Arabia; Hubert Department of Global Health, Rollins School of Public Health, Emory University, Atlanta, GA, USA; Al-Faisal University, Riyadh, Saudi Arabia; Al-Faisal University, Riyadh, Saudi Arabia; Department of Gastroenterology and Hepatology, Erasmus MC-University Medical Center, Rotterdam, the Netherlands; WHO Collaborating Centre for Travellers' Health, Institute for Epidemiology, Biostatistics and Prevention, University of Zürich Centre for Travel Medicine, MilMedBiol Competence Centre, University of Zürich, Switzerland

Asymptomatic infection refers to a situation when a person is infected with a virus but never develop any symptoms. A pre-symptomatic individual, on the other hand, is an infected person who shows no symptoms at the time of testing but later develops symptoms. Asymptomatic infection with the Severe Acute Respiratory Syndrome Coronavirus (SARS-CoV) was rarely reported [1], whereas this situation has been well-described in relation to the Middle East Respiratory Syndrome Coronavirus (MERS-CoV). There are three patterns of transmissions for MERS-CoV including camel-to-human transmission, healthcare associated transmission and community transmission [2]. However, healthcare-associated transmission is a hallmark of this virus [3] and the detection rate of asymptomatic individuals was shown to increase with expanded testing and screening [4]. In a systematic review, the rate of detection of asymptomatic infection with MERS-CoV increased from 0% to 28.6% over time in parallel with increased testing [5,6]. It is estimated that asymptomatic cases constitute about 10% of the total number of MERS cases [5]. In an analysis of large numbers of cases from 2012 to 2018, the rate of asymptomatic infection was 24.3% (n = 266/1094) among secondary cases vs. 1.4% (n = 9/642) among primary cases (p < 0.001) [7]. In addition, the rates of asymptomatic MERS were variable among different populations and these were 1% among contacts of healthcare workers and 42% of hospital admissions [6]. Rates of asymptomatic MERS between 0.5 and 0.8% of screened contacts [8,9] and 12.5–81.8% among confirmed cases [[10], [11], [12], [13], [14], [15]] (Table 1).

**Table 1:** **A summary of studies addressing asymptomatic MERS and SARS-CoV-2 cases in Humans based on PCR tests**.

Year	Proportion of asymptomatic cases among confirmed cases (%)	Number of confirmed cases among asymptomatic screened contacts	Reference
MERS			
2012–2013	18/144 (12.5%)	–	[8]
2012–2013	–	4/520 (0.8%)	[9]
2015	–	3/591 (0.5%)	[10]
2014	64/255 (25.1%)	–	[11]
2012–2013	9/11 (81.8%)	–	[12]
2012–2016	13/31 (41.9%)	–	[13]
2014–2016	3/7 (42.8%)	–	[14]
2012–2018	266/1094 (24.3%) among secondary		[7]
2012–2018	9/642 (1.4%) among primary cases		[7]
SARS-CoV-2			
up to 30 April 2020	873/2788 (48.2%) among confirmed cases		[16]
February 4, 2021	11, 069/19,884 (55.6%) among confirmed	(0.038%) 11,516 asymptomatic of all 29,776,306 tested	[17]
January 2020–April 2021	35.1% (95% CI: 30.7–39.9%) remain asymptomatic		[18]

The infection course of SARS-CoV-2 has been extensively studied and the spectrum of the disease ranges from asymptomatic to mild, moderate, and severe disease [19]. In a two-point survey in a skilled nursing facility, 27 of 48 (56%) of persons tested were asymptomatic at the time of testing and of these, 24 subsequently developed symptoms with a median time to onset of 4 days [20]. The rate of asymptomatic SARS-CoV-2 among confirmed cases was reported to range from 35.1% to 55.6% [[16], [17], [18]]. On the other hand, a systematic analysis reported that the rate of asymptomatic infection was 0.038% (11,516 of 29,776,306 tested individuals) [17]. The rate of asymptomatic persons was variable among different contacts as follow: 39% in male, 56% in female and 50% in children [16] and an overall rate of 24.51% (95% CI: 14.38, 36.02) among contacts [21]. However, it is important to recognize the contribution of asymptomatic infections to the risk of transmission. Screening for and finding asymptomatic cases also depends on the type of testing used. Screening with Reverse Transcriptase Polymerase Chain Reaction (RT-PCR), is more likely to detect asymptomatic cases compared to screening with Rapid Antigen Tests (RATs) [22]. A systematic review of 130 studies showed that secondary attack rate in contacts of asymptomatic vs. symptomatic infection was 0.32 (95% CI 0.16–0.64) [23]. Another systematic review showed a risk of 0.35 (95% CI 0.1–1.27) for asymptomatic and 0.63 (95% CI 0.18–2.26) for pre-symptomatic people compared to symptomatic individuals [24]. The risk of transmission varied among different groups of contacts: familial clusters (15.72%), adults (29.48%), children (24.09%), and healthcare workers (0%) [21].

Further studies examined the differences in viral Ct values between different groups. In one study, the median Ct values of asymptomatic, pre-symptomatic, atypical symptoms, and typical symptoms were similar [20]. Another study showed similar values at enrollment but lower than symptomatic in the first 19 days [25], however, this is an unusual finding as lower CT-values indicated higher viral loads and tend to be seen more in symptomatic patients. The mean time in days to negative PCR tests in different studies showed different results: similar [26], longer duration of viral shedding than symptomatic group [27], and faster viral clearance [25]. These differences are probably related to the different study designs and the small number of patients. The SARS-CoV-2 virus-specific IgG levels in the asymptomatic group were significantly lower compared to those in symptomatic patients [27]. In addition, a set of pro- and anti-inflammatory cytokines were elevated in symptomatic patients compared to asymptomatic persons [27].

The contribution of asymptomatic individuals with different human coronaviruses to transmission cycles, outbreaks and pandemics requires further research. Future studies should carefully examine the longitudinal clinical course of those individuals, the viral dynamics, viral loads and contribution to transmission. Furthermore, factors such as vaccination status and different variants of the virus should be taken into consideration. It had been noted that a good proportion of asymptomatic SARS-CoV-2 had abnormal radiographic or laboratory data [28] raising the question about true asymptomatic or being pre-symptomatic. In addition, PCR testing had showed intermittent positivity with prolonged positive intervals [29,30] and these cases may not reflect true infectivity. Thus, de-isolation strategy had evolved the course of the COVID-19 pandemic from PCR-based, to symptom-based, to time-based isolation [31].
